# The predation relationship between online medical search and online medical consultation—empirical research based on Baidu platform data

**DOI:** 10.3389/fpubh.2024.1392743

**Published:** 2024-08-29

**Authors:** Yang Wang, Lingshi Ran, Wei Jiao, Yixue Xia, Yuexin Lan

**Affiliations:** Research Center for Network Public Opinion Governance of CPPU, Langfang, China

**Keywords:** online medical search, online medical consultation, health information needs, health information-seeking behavior, evolutionary patterns

## Abstract

**Introduction:**

This study investigates the mutual influence between online medical search and online medical consultation. It focuses on understanding the health information needs that drive these health information-seeking behaviors by utilizing insights from behavioral big data.

**Methods:**

We used actual behavioral data from Chinese internet users on Baidu platform’s “Epidemic Index” from November 26, 2022, to January 25, 2023. Data modeling was conducted to ensure the reliability of the model. Drawing on the logistic model, we constructed a foundational model to quantify the evolutionary patterns of online medical search and online medical consultation. An impact function was defined to measure their mutual influence. Additionally, a pattern detection experiment was conducted to determine the structure of the impact function with maximum commonality through data fitting.

**Results:**

The analysis allowed us to build a mathematical model that quantifies the nonlinear correlation between online medical search and online medical consultation. Numerical analysis revealed a predation mechanism between online medical consultation and online medical search, highlighting the role of health information needs in this dynamic.

**Discussion:**

This study offers a novel practical approach to better meet the public’s health information needs by understanding the interplay between online medical search and consultation. Additionally, the modeling method used here is broadly applicable, providing a framework for quantifying nonlinear correlations among different behaviors when appropriate data is available.

## 1 Introduction

The COVID-19 pandemic has resulted in significant human losses and widespread disruption to economic, social, and health systems worldwide (1). Due to safety concerns and the convenience they offer, online healthcare services have emerged as viable alternatives to traditional in-person medical visits during the pandemic (2). As public access to health information has increasingly moved online, we can now leverage big data from online healthcare platforms to study internet users’ health information-seeking behaviors and gain deeper insights into their health information needs.

**Figure 1:**
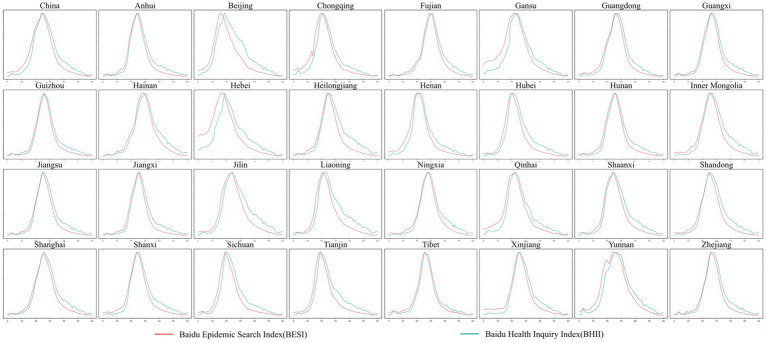
Nationwide and provincial-level administrative region big data on internet user behavior with available statistical data.

The online health information behavior of internet users generates substantial amounts of electronic data. Digital tools such as Google Trends and Baidu Index track online medical search, reflecting the degree of internet users’ search interest. These data form the behavioral big data of internet users (3), proven effective and possessing predictive value, and are widely applied in studying changes in internet users’ interests and evaluating human behavior (4). Behavioral data for online medical consultation, typically compiled by various online healthcare service providers (2), indicate the number of individuals seeking medical advice through their platforms. For COVID-19, Baidu introduced an “Epidemic Index” similar to the Baidu Index. The “Epidemic Index” compiles data from 31 provincial-level administrative regions in China, excluding Hong Kong, Macau, and Taiwan. Within this index, the “Baidu Epidemic Search Index (BESI)” is generated based on internet users’ online medical search behavior, reflecting the level of attention and ongoing changes in searches for COVID-19 symptoms, epidemic prevention materials, and related needs. The “Baidu Health Inquiry Index (BHII)” is generated based on online medical consultation behavior, synthesizing the scale of local internet users’ inquiries on epidemic prevention and COVID-19 treatment on Baidu Health. This study analyzed data from November 26, 2022, to January 25, 2023, for each provincial-level administrative region with available statistics and the aggregated national data. The findings revealed correlations in both BESI and BHII across regions. Figure 1 illustrates the behavioral big data of internet users at the national level and in provinces with available statistics. This time frame coincides with a complete natural evolution cycle of the epidemic, from emergence to outbreak and eventual decline. On December 7, 2022, the Joint Prevention and Control Mechanism of the State Council of China introduced the “Notice on Further Optimizing and Implementing Measures for COVID-19 Prevention and Control,” commonly referred to as the “10 New Measures” to optimize the COVID-19 response (5). The relative relaxation of control policies inevitably led to a surge in new coronavirus infections across regions. Subsequently, due to robust social security and healthcare services, the number of new coronavirus infections gradually decreased.

**Figure 2:**
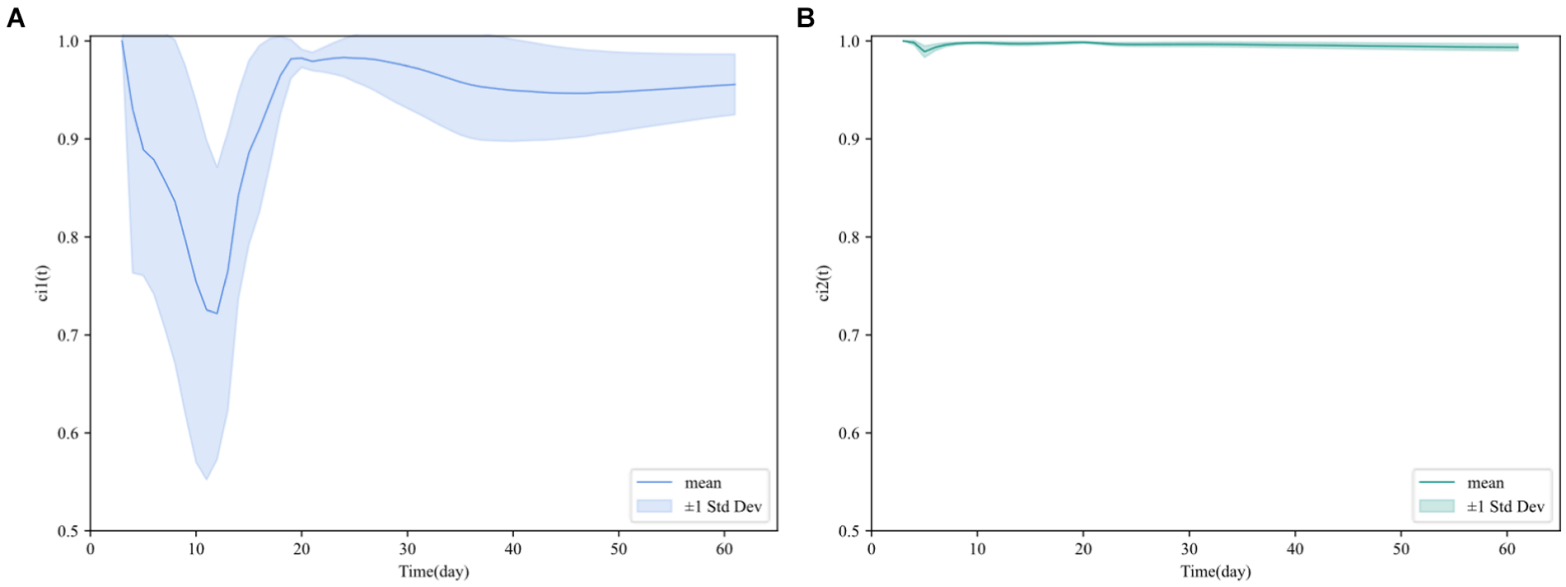
**(A)** Dynamic correlation coefficient ci1 (T). **(B)** Dynamic correlation coefficient ci2 (t).

To explore the relationship between BESI and BHII, this study conducted dynamic correlation analyses on 32 datasets, comprising national data and data from provincial-level administrative regions with available statistics and their corresponding cumulative data. The use of cumulative data is justified by the distinct characteristics of evolution and variation. Evolution emphasizes temporality and historical influences, while variation highlights the current state and spatial orientation. Since current statistical data often lack historical information, we cumulated the data to incorporate historical influences into the analysis. Cumulative data allow us to capture evolving trends over time, facilitating a more comprehensive understanding of the evolutionary processes and trends in the data, thus providing more accurate models and analysis results. As correlation analysis requires a minimum of three data points, we initiated the analysis for each region from 3 days and incrementally adding 1 day’s worth of data for correlation analysis. We focused on the strength of correlation, temporarily disregarding its direction. Therefore, dynamic correlation coefficients, denoted as $c_{i1}\left(t\right)$ for daily data and $c_{i2}\left(t\right)$ for cumulative data, were calculated by taking the absolute value of the Pearson correlation coefficient between BESI and BHII. Here, i=1,2,3,……,32 , corresponding to the 32 datasets. The computational results indicate a significant correlation between BESI and BHII, particularly in terms of the dynamic correlation observed in the cumulative data. Thus, it is necessary to explore the relationship between BESI’s cumulative data and BHII’s cumulative data as variables. To demonstrate the overall trend and variability range of data correlations, we calculated the mean and standard deviation at each time point. Figure 2 shows the mean line and the shaded area representing the standard deviation, indicating the range of variability.

This study focuses on exploring the correlation between online medical consultation and online medical search, highlighting its significant research value. Theoretically, the model constructed in this research quantifies the nonlinear correlation between these two health information-seeking behaviors during an epidemic outbreak. Analysis of the model reveals a predation mechanism between online medical consultation and online medical search, which we interpret as driven by the demand for health information. Moreover, the data modeling approach employed in this study is both innovative and broadly applicable, capable of examining nonlinear correlations between other behaviors. By selecting appropriate behavioral data as variables for data modeling, one can gain insights into the underlying patterns of mutual influence among different behaviors.

Practically, this study leverages big data on internet users’ behaviors to gain insights into their health information-seeking actions and analyze their health information needs, which holds significant value for enhancing health communication. Both online medical search and online medical consultation are fundamentally health information-seeking behaviors. Online medical search involves using search engines to locate medical information online (6). For many individuals, this is the initial step in understanding symptoms, diseases, and health information (7). Online medical consultation refers to internet-based remote consultations between patients and doctors, serving as a vital means for users to obtain health information from healthcare professionals (8). Information needs drive information behaviors (9). From this perspective, internet users’ health information-seeking behavior reflects their health information needs. Many people turn to the internet to fulfill their health information needs (10, 11). Summarizing users’ overall information needs and their specific demands for online health information is essential for producing content that meets their needs and is the only way to ensure the provision of high-quality information (12).

The structure of this paper is outlined as follows. Section 2 provides a review of relevant studies. Section 3 details the construction and validation of the foundational model. Based on the evolutionary mechanisms of epidemics and online medical behaviors, the foundational model in this section is constructed by drawing on the logistic model. Its applicability is verified through data fitting. Section 4 focuses on nonlinear correlation modeling. This section introduces the exploratory experiments designed for this study, and based on the experimental results, identifies the most suitable structural factors. Consequently, a nonlinear correlation model quantifying the mutual influence between online medical search and online medical consultation is developed, building upon the foundational model. Section 5 is dedicated to the discussion, interpreting the model mechanisms and analyzing the health needs reflected in health information-seeking behavior. Section 6 serves as the conclusion, summarizing the findings of this research.

## 2 Literature review

### 2.1 Health information needs

Information needs refer to the “recognition that your knowledge is inadequate to satisfy your specific goals” (13). Investigating users’ information needs is beneficial for designing more effective information services (14). The study of health information needs has garnered widespread attention from scholars across various disciplines, particularly in health sciences such as nursing and medicine (15). Existing research has produced fruitful outcomes, each with its unique context (16–18). Numerous studies have explored the information needs of different user groups in diverse contexts through various approaches and methods (15), often focusing on specific populations and lacking comprehensive research covering diverse demographics. For instance, Qian & Gui conducted a study on health-related posts extracted from online communities, employing text classification through word frequency analysis to identify health information needs among older adult online community users (19). Dau et al. surveyed rectal cancer patients to assess how to meet their health information needs throughout the entire care process (20). Li et al. collected data through semi-structured interviews and surveys, exploring the nature of mental health information needs among Chinese students and identifying online resources they use to fulfill these needs (21). However, most previous studies on health information needs and seeking behaviors relied on subjective reports from users, predominantly using surveys and interviews, with limited utilization of objective measurement methods (22), and few employed big data analytics. Moreover, past research has identified significant variations in health information-seeking behavior, necessitating further classification of information needs and sources (23). However, there is a lack of research on the interrelations among different health information-seeking behaviors and the specific information needs each behavior represents. Addressing these gaps, this study utilizes behavioral big data, specifically the “Epidemic Index” of Chinese internet users, as research data to explore the health information needs reflected in various health information-seeking behaviors.

### 2.2 Online health information seeking behavior

Health information-seeking behavior refers to the process of gathering health knowledge stemming from health queries or needs (24). During the pandemic, governments worldwide imposed restrictions on individual freedoms by enforcing stay-at-home orders, permitting office work only under certain circumstances, and implementing strict “physical distancing” rules to prevent the spread of COVID-19 (25). Online medical consultation have been emphasized to prevent cross-infections or adhere to the social distancing measures encouraged by governments, leading an increasing number of patients to opt for online medical consultation as an alternative to traditional healthcare services (26). Simultaneously, the outbreak of COVID-19 has resulted in a surge of individuals globally searching for health information on the internet (27). This study focuses on two health information-seeking behaviors: online medical search and online medical consultation.

Online medical search is a behavior driven by information needs and responsive to information needs (2). As early as 2004, MORAHAN-MARTIN conducted a review of how internet users seek, evaluate, and utilize online health information (28). The quality of online medical information has been a consistent focus of scholarly attention. Existing literature employs various terms to describe the concept of quality, including but not limited to quality, credibility, trust, reliability, dependability, and utility (29). Comparative studies on multiple search engines have been conducted (30), assessing the quality of various surgical procedures, diseases, and healthcare information (31, 32). As online health information becomes increasingly popular among patients, concerns have emerged regarding the impact of patients’ internet health information-seeking behavior on their relationships with healthcare professionals (33). Furthermore, the psychological and behavioral aspects of internet users are also a focal point of research. For example, numerous scholars have investigated the relationship between online medical search and cyberchondria (34). Eysenbach & Köhler explored the retrieval and evaluation techniques consumers employ when searching for health information online (35). Thiessen et al. studied the reasons why cancer patients turn to the internet for information (36).

Patient-centered care and/or medicine have been central to the healthcare agenda for several decades (37). In recent years, research on online medical consultation has been predominantly patient-centric (38). For instance, Yang et al. investigated how doctors’ online consultation behaviors, viewed through the lens of trust development, reduce patient switching and enhance patient satisfaction (39). Sandeep et al. surveyed to examine external and internal stimuli influencing users’ reactions to online doctor consultation platforms (40). Utilizing records of online medical consultation, Cao et al. explored the multilayered factors influencing patient proactiveness in online medical consultation (26).

In addition to studying the behavior of online medical information-seeking itself, many scholars have recognized the predictive value of monitoring behavioral data generated by online medical search and consultation (2). Google Flu Trends was among the earliest studies to apply big data from search behavior. Ginsberg et al. used Google web search logs to create a model for influenza monitoring (41). In recent years, such data from online medical search have also been widely utilized in research. For instance, Li et al. assessed the predictive value of internet search data, including sources from search engines and social media, for the outbreak of COVID-19 in China, finding that utilizing the Sina Weibo Index, Google Trends, and Baidu Index could accurately and timely predict the onset and progression of the pandemic (42). Huang et al., in their research, focused on various behavioral data, evaluating the predictive value of online medical consultation, appointments, and searches for regional outbreaks of COVID-19 (2).

Many scholars have conducted extensive research on online health-seeking behavior, yielding significant results. Previous studies have demonstrated the effectiveness and predictive value of behavioral data generated by online medical activities. However, no scholars have investigated the mutual influence between two distinct online health information-seeking behaviors: online medical search and online medical consultation. Additionally, past research on epidemic monitoring using behavioral data has predominantly employed regression models, which represent a relatively singular methodology. This study, through the analysis of behavioral big data, identifies a strong correlation between online medical search and online medical consultation. Consequently, it employs a non-linear correlation modeling approach, using BESI and BHII to explore patterns, construct a model quantifying the mutual impact between online medical search and online medical consultation, and investigate the relationship between the two.

## 3 Fundamental model

In this chapter, the mutual influence between online medical search and online medical consultation is temporarily excluded. Instead, we propose a foundational model to describe the cumulative data quantity changes in BESI and BHII, subsequently validating the accuracy of this foundational model. Before constructing the model, it is essential to clarify several key assumptions made during the model construction and data analysis process in this study: 1. BESI and BHII can quantify the two health information-seeking behaviors, online medical search and online medical consultation, and can be used to study the mutual influence between them. This is based on Baidu’s description of BESI and BHII, which states that these indices can quantify online medical search and online medical consultation under unified data specifications and rules, reflecting the objective patterns of these health information acquisition behaviors. These modeling assumptions are scientifically sound. 2. The evolution of an epidemic follows a life cycle, which can be roughly divided into the rising period, rapid transmission period, and transmission saturation period (43). The scale evolution of online medical search and online medical consultation is consistent with the epidemic’s evolution and also follows a life cycle. Therefore, the foundational model can be established by referring to the modeling principles of the Logistic model. 3. There is a mutual influence between the two health information-seeking behaviors, online medical search and online medical consultation. To model the interaction between them, it is necessary to improve the basic model by introducing interaction terms.

### 3.1 Constructing the foundation model

Assuming that the cumulative quantities of BESI and BHII are continuous and differentiable functions with respect to time t , denoted as $x_{i1}\left(t\right)$ and $x_{i2}\left(t\right)$ respectively. Due to the existence of a lifecycle, both BESI and BHII cumulative quantities have upper limits. Let $K_{i1}$ represent the upper limit of BESI and $K_{i2}$ denote the upper limit of BHII. The remaining space for BESI is given by $\left(1-\frac{x_{i1}}{K_{i1}}\right)$ , and for BHII, it is $\left(1-\frac{x_{i2}}{K_{i2}}\right)$ . Neglecting the mutual influence between them, their growth rates are directly proportional to their current sizes and the remaining space, respectively.

Based on the above analysis, we construct a foundational model to quantify the cumulative changes in BESI and BHII:


$$\left\{ \begin{array}{c} \frac{dx_{i1}}{dt}=r_{i1}x_{i1}\left(1-\frac{x_{i1}}{K_{i1}}\right)\\ \frac{dx_{i2}}{dt}=r_{i2}x_{i2}\left(1-\frac{x_{i2}}{K_{i2}}\right) \end{array}\right.$$

Here, i=1,2,3,….,32 , represents 32 distinct sets of data, where $x_{i1}$ is the function quantifying the cumulative changes in BESI, with an initial value of $x_{i1}\left(0\right)$ . Similarly, $x_{i2}$ is the function quantifying the cumulative changes in BHII, with an initial value of $x_{i2}\left(0\right)$ . The parameters $r_{i1}>0$ and $r_{i2}>0$ denote the inherent growth rates for BESI and BHII cumulative indices, respectively. $K_{i1}$ represents the upper limit for BESI cumulative quantity, while $K_{i2}$ represents the upper limit for BHII cumulative quantity.

### 3.2 Validating the foundation model

Before proceeding with further investigations, it is imperative to ensure the accuracy of our foundational assumptions. To this end, we employ the foundational model to fit the data and validate its integrity. The methodology for parameter estimation follows the differential regression approach outlined in the work of Lan et al. (44). The specific steps are as follows:

Firstly, we transform the differential equations in the foundational model into a discrete form, namely:


$$\left\{ \begin{array}{c} \Delta x_{i1}\left(n\right)=r_{i1}x_{i1}\left(1-\frac{x_{i1}}{K_{i1}}\right)=r_{i1}x_{i1}-\frac{r_{i1}}{K_{i1}}{x_{i1}}^{2}\\ \Delta x_{i2}\left(n\right)=r_{i2}x_{i2}\left(1-\frac{x_{i2}}{K_{i2}}\right)=r_{i2}x_{i2}-\frac{r_{i2}}{K_{i2}}{x_{i2}}^{2} \end{array}\right.$$

In this context, $\Delta x_{i1}\left(n\right)=x_{i1}\left(n\right)-x_{i1}\left(n-1\right)$ , and $\Delta x_{i2}\left(n\right)=x_{i2}\left(n\right)-x_{i2}\left(n-1\right)$ . Here, $\Delta x_{i1}\left(n\right)$ and $\Delta x_{i2}\left(n\right)$ represent the statistical data for BESI and BHII at time n , while $x_{i1}\left(n\right)$ and $x_{i2}\left(n\right)$ represent the cumulative data for BESI and BHII at the same time. Each variable in the difference equation can be obtained through statistical and computational methods. With these transformations, the parameter fitting problem of the differential equations is converted into a regression analysis problem of the discrete equations. $\Delta x_{i1}\left(n\right)$ exhibits a bivariate linear relationship with $x_{i1}$ and ${x_{i1}}^{2}$ , while $\Delta x_{i2}\left(n\right)$ shows a bivariate linear relationship with $x_{i2}$ and ${x_{i2}}^{2}$ . By applying bivariate linear regression analysis, regression coefficients such as $r_{i1}$ , $-\frac{r_{i1}}{K_{i1}}$ , $r_{i2}$ , and $-\frac{r_{i2}}{K_{i2}}$ can be obtained. Consequently, the model parameters $r_{i1}$ , $K_{i1}$ , $r_{i2}$ , and $K_{i2}$ can be derived.

To validate the accuracy of the foundational model, this study conducted a fitting analysis of the complete cycle data of 32 sets of corresponding cumulative quantities for BESI and BHII, totaling 64 regression analyses. Three criteria, namely structural testing, correlation testing, and significance testing, were established to assess the results of the model fitting, as presented in Table 1.

**Table 1:** Three aspects criteria for evaluating parameter estimates.

Structural Testing	The parameter values conforming to the model assumptions, with the evolutionary equation for BESI ( $r_{i1}>0$ , $-\frac{r_{i1}}{K_{i1}}<0$ ), are considered to pass the structural testing. Similarly, for BHII, the evolutionary equation conditions ( $r_{i2}>0$ , $-\frac{r_{i2}}{K_{i2}}<0$ ) are regarded as passing the structural testing.
Correlation Testing	If the coefficient of determination ( R−squared ) for each regression analysis is greater than 0.9, it indicates that the model error is small and is considered to pass the correlation test.
Significance Testing	If the P−value for all variables in the regression analysis is less than 0.05, it indicates that the regression coefficients are highly significant, and the model is considered to pass the significance test.

The results indicate that, in the equation modeling the variation in BHII cumulative quantity, the fitting performance is relatively poorer for only 8 sets of data. However, even for these 8 sets of data, the minimum R−squared value is 0.79, indicating a strong correlation. All other regression analyses meet the criteria set for the structural, correlation, and significance tests. The high degree of fit between the data and the baseline model is evident from these results. Therefore, it can be concluded that the underlying assumptions in this study are reasonable, and the baseline model constructed is accurate.

## 4 Nonlinear correlation modeling

Upon the foundation of the baseline model, functions $f_{1}$ and $f_{2}$ are introduced to quantify the influence of BHII cumulative quantity variations on the growth rate of BESI cumulative quantity and the influence of BESI cumulative quantity variations on the growth rate of BHII cumulative quantity, respectively. These functions, referred to as impact functions, are assumed to be solely dependent on $x_{i1}$ and $x_{i2}$ , as this study posits that the patterns of mutual influence between online medical search and online medical consultation remain constant over time. Taking into account the reciprocal impact between the two health-seeking behaviors, the model for quantifying the variations in BESI and BHII cumulative quantities becomes:


$$\left\{ \begin{array}{c} \frac{dx_{i1}}{dt}=r_{i1}x_{i1}\left(1-\frac{x_{i1}}{K_{i1}}\right)+f_{i1}\left(x_{i1},x_{i2}\right)\\ \frac{dx_{i2}}{dt}=r_{i2}x_{i2}\left(1-\frac{x_{i2}}{K_{i2}}\right)+f_{i2}\left(x_{i1},x_{i2}\right) \end{array}\right.$$

What do $f_{1}\left(x_{i1},x_{i2}\right)$ and $f_{2}\left(x_{i1},x_{i2}\right)$ specifically entail? This is what we need to delve into.

### 4.1 Structural analysis of the influence function

The structure of $f_{1}\left(x_{i1},x_{i2}\right)$ and $f_{2}\left(x_{i1},x_{i2}\right)$ is the first aspect to determine. In the foundational model, the rate of change of BESI and BHII cumulative quantities is described by the differential equation $\frac{dx}{dt}=rx\left(1-\frac{x}{K}\right)=rx-\frac{r}{K}x^{2}$ , where the rate of change $\frac{dx}{dt}$ is a linear function concerning x and $x^{2}$ , with the highest order of the variable being quadratic. Consequently, in $f_{1}\left(x_{i1},x_{i2}\right)$ and $f_{2}\left(x_{i1},x_{i2}\right)$ , the highest order of a single variable can only be quadratic. Therefore, $f_{1}\left(x_{i1},x_{i2}\right)$ may include at most six structural factors: $x_{i2}$ , ${x_{i2}}^{2}$ , $x_{i1}x_{i2}$ , $x_{i1}{x_{i2}}^{2}$ , ${x_{i1}}^{2}x_{i2}$ , and ${x_{i1}}^{2}{x_{i2}}^{2}$ . In other words, $f_{1}\left(x_{i1},x_{i2}\right)$ might consist of one, two, …, or all six of the aforementioned structural factors. Furthermore, the positive or negative sign of the coefficients for these structural factors needs consideration; each coefficient has two possible signs. Consequently, $f_{1}\left(x_{i1},x_{i2}\right)$ has $C\begin{array}{c} 1\\ 6 \end{array}*2+C\begin{array}{c} 2\\ 6 \end{array}*2^{2}+C\begin{array}{c} 3\\ 6 \end{array}*2^{3}+C\begin{array}{c} 4\\ 6 \end{array}*2^{4}+C\begin{array}{c} 5\\ 6 \end{array}*2^{5}+C\begin{array}{c} 6\\ 6 \end{array}*2^{6}=668$ possible structures. Similarly, $f_{2}\left(x_{i1},x_{i2}\right)$ encompasses at most the same six structural factors, yielding the same 668 possible structures.

### 4.2 Pattern detection

The structures of $f_{1}\left(x_{i1},x_{i2}\right)$ and $f_{2}\left(x_{i1},x_{i2}\right)$ differ, corresponding to distinct differential equations governing the growth patterns of quantified BESI and BHII cumulative quantities. To achieve the research objective of extracting common patterns from the data, we propose a pattern detection experiment. The general approach is to employ all possible differential equations to fit the data from 32 groups, determining the most suitable structure for the impact functions.

**Figure 3:**
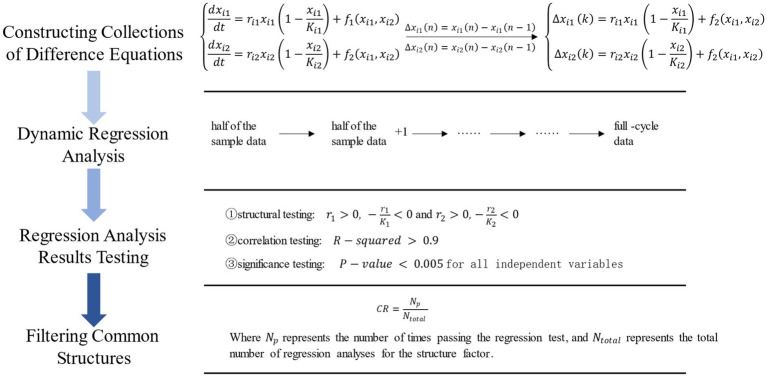
Schematic diagram of pattern detection experiment.

The specific experimental procedure is illustrated in Figure 3. The detailed steps involve:

1. Constructing a set of difference equations: transform the evolutionary models of BESI and BHII into their corresponding difference equations:
$$\left\{ \begin{array}{c} \Delta x_{i1}\left(n\right)=r_{i1}x_{i1}\left(1-\frac{x_{i1}}{K_{i1}}\right)+f_{2}\left(x_{i1},x_{i2}\right)\\ \Delta x_{i2}\left(n\right)=r_{i2}x_{i2}\left(1-\frac{x_{i2}}{K_{i2}}\right)+f_{2}\left(x_{i1},x_{i2}\right) \end{array}\right.$$

Forming two sets of difference equations, $S_{1}$ and $S_{2}$ , each containing 63 equations. Here, $\Delta x_{i1}\left(n\right)=x_{i1}\left(n\right)-x_{i1}\left(n-1\right)$ and $\Delta x_{i2}\left(n\right)=x_{i2}\left(n\right)-x_{i2}\left(n-1\right)$ , where n=1,2,3,….,32 .

2. Dynamic regression analysis: A robust mathematical model should provide predictive value, demonstrating excellent fitting performance not only on full-cycle data but also on datasets of varying sizes. Therefore, for each equation in sets $S_{1}$ and $S_{2}$ , dynamic regression analysis is conducted on the 32 data sets. For each equation, regression analysis begins with half of the data and gradually increases by one data point until the full-cycle data is reached.
3. Regression testing: After performing regression analysis for each equation in sets $S_{1}$ and $S_{2}$ , regression testing is conducted according to the standards set in Section 3.2. The criteria include structural, correlational, and significance aspects, as shown in Table 1. If the results meet the criteria in all three aspects, the equation is considered to pass the regression test.
4. Selection of common structures: To filter out structures with the maximum commonality, a Conformity Rate (CR) is defined. CR describes the percentage of times a structure, through dynamic regression analysis and with consistent positive or negative coefficients, passes the three tests. The structure with the highest conformity rate clearly exhibits the maximum commonality.
$$CR=\frac{N_{p}}{N_{\mathrm{total}}}$$

Where $N_{p}$ represents the number of passes in regression tests, and $N_{\mathrm{total}}$ is the total number of times the structure undergoes differential regression.

### 4.3 Nonlinear correlation model

**Figure 4:**
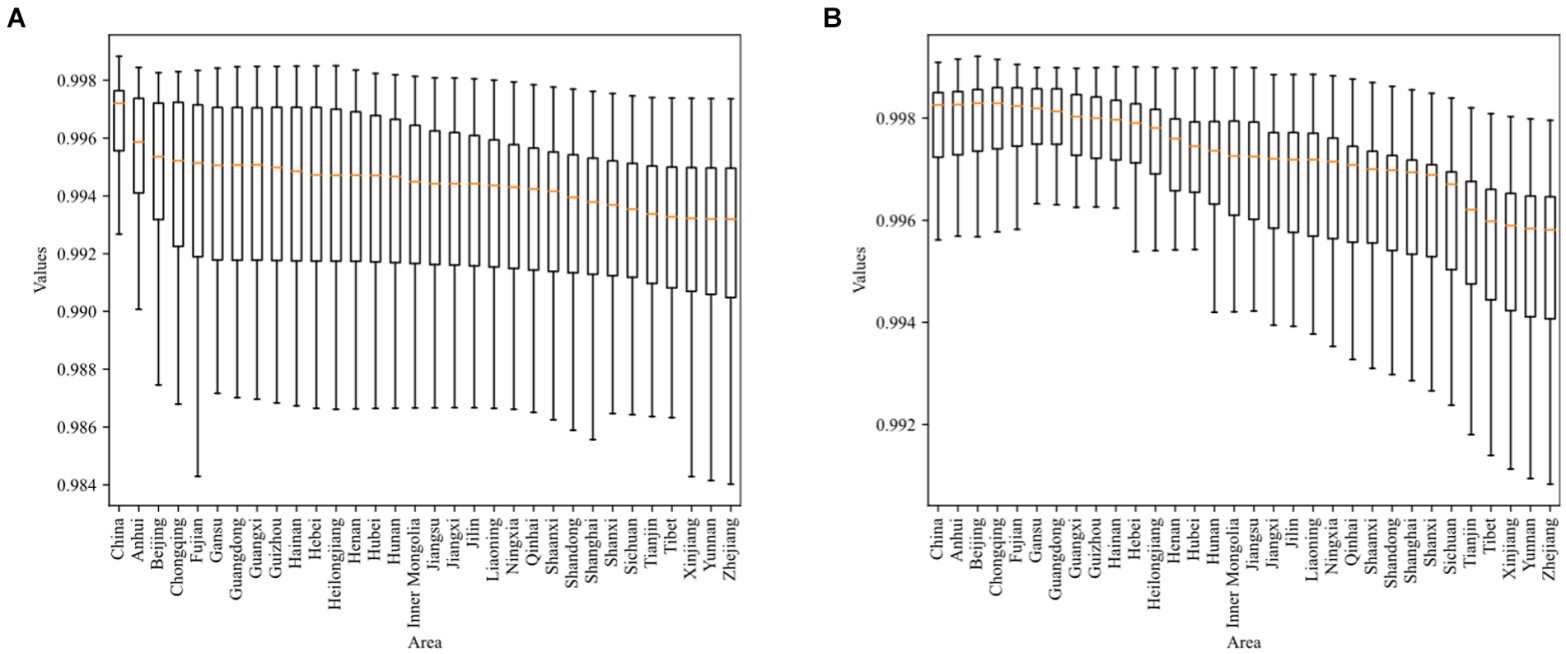
**(A)** R-Squared distribution for the first equation in dynamic regression analysis. **(B)** R-squared distribution for the second equation in dynamic regression analysis.

**Figure 5:**
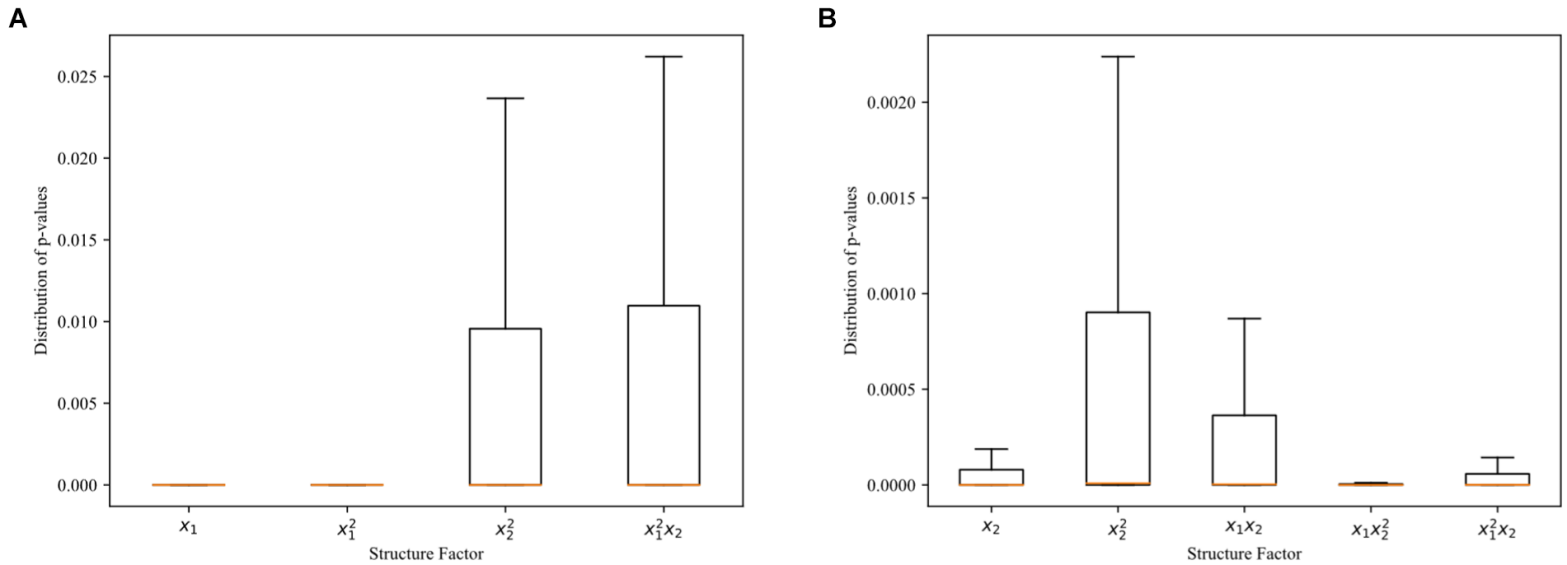
**(A)** Distribution of *p*-values for the first equation in dynamic regression analysis. **(B)** Distribution of *p*-values for the second equation in dynamic regression analysis.

The results of the pattern detection experiment reveal that $f_{1}\left(x_{i1},x_{i2}\right)=a_{1}{x_{i2}}^{2}+a_{2}{x_{i1}}^{2}x_{i2}$ with $a_{1}>0$ and $a_{2}<0$ is the most common structure among the 668 possible structures for $f_{1}\left(x_{i1},x_{i2}\right)$ , with a conformity rate of 73.44%. Similarly, $f_{2}\left(x_{i1},x_{i2}\right)=b_{1}x_{i1}x_{i2}+b_{2}x_{i1}{x_{i2}}^{2}+b_{3}{x_{i1}}^{2}x_{i2}$ with $b_{1}>0$ , $b_{2}>0$ , and $b_{3}<0$ is the most common structure among the 668 possible structures for $f_{2}\left(x_{i1},x_{i2}\right)$ , with a conformity rate of 80.18%. We recorded the R−squared and P−values for each regression analysis and grouped them by region to create box plots illustrating the distribution of R−squared values (Figure 4). Additionally, box plots grouped by structure factors were generated to illustrate the distribution of p−values for each structure factor (Figure 5). These visualizations demonstrate a high level of model fit, with significant values for each structure factor.

Based on the experimental results, we establish a model to quantify the nonlinear correlation between online medical search and online medical consultation:


$$\left\{ \begin{array}{c} \frac{dx_{1}}{dt}=r_{1}x_{1}\left(1-\frac{x_{1}}{K_{1}}\right)+a_{1}{x_{2}}^{2}+a_{2}{x_{1}}^{2}x_{2}\\ \frac{dx_{2}}{dt}=r_{2}x_{2}\left(1-\frac{x_{2}}{K_{2}}\right)+b_{1}x_{1}x_{2}+b_{2}x_{1}{x_{2}}^{2}+b_{3}{x_{1}}^{2}x_{2} \end{array}\right.$$

The symbols and their meanings involved in the above model are presented in Table 2.

**Table 2:** Model parameters and their meanings.

Parameter	Meaning
t	Time.
$x_{1}\left(t\right)$	Quantitative function representing the cumulative quantity changes of BESI, with the initial value denoted as $x_{10}$ .
$x_{2}\left(t\right)$	Quantitative function representing the cumulative quantity changes of BHII, with the initial value denoted as $x_{20}$ .
$r_{1}$	Intrinsic growth rate of cumulative data for BESI.
$r_{2}$	Intrinsic growth rate of cumulative data for BHII.
$K_{1}$	The upper limit of cumulative data for BESI.
$K_{2}$	The upper limit of cumulative data for BHII.
$a_{1},a_{2}$	Structural factor coefficients of the growth rate impact function of cumulative BHII on cumulative BESI, where $a_{1}>0$ and $a_{2}<0$ .
$b_{1},b_{2},b_{3}$	Structural factor coefficients of the growth rate impact function of cumulative BESI on cumulative BHII, where $b_{1}>0$ , $b_{2}>0$ , and $b_{3}<0$ .

## 5 Discussion

### 5.1 Explanation of model mechanism

The model constructed in this study is derived from an in-depth analysis of large-scale behavioral data, revealing the nonlinear correlations present in the real world. Unlike other mathematically simplified models, the regularities of this model are not solely concealed within its equation structure. Merely by observing the equation structure, it becomes challenging to interpret the underlying correlations between online medical search and consultation. To explore the potential underlying principles, a thorough analysis of this intricate and chaotic nonlinear structure is imperative.

Firstly, we need to ascertain the mechanism of mutual influence between online medical search and online medical consultation. In the model, this is manifested in the impact of $x_{2}$ on $\frac{dx_{1}}{dt}$ and the influence of $x_{1}$ on $\frac{dx_{2}}{dt}$ . Each term in $f_{1}\left(x_{1},x_{2}\right)$ contains $x_{2}$ , and each term in $f_{2}\left(x_{1},x_{2}\right)$ contains $x_{1}$ , precisely validating our model’s ability to quantify the mutual influence between the two. We transform the bidirectional interaction model of online search and consultation into:


$$\left\{ \begin{array}{c} \frac{dx_{1}}{dt}=r_{1}x_{1}\left(1-\frac{x_{1}}{K_{1}}\right)+\varphi_{1}x_{2}\\ \frac{dx_{2}}{dt}=r_{2}x_{2}\left(1-\frac{x_{2}}{K_{2}}\right)+\varphi_{2}x_{1} \end{array}\right.$$

**Figure 6:**
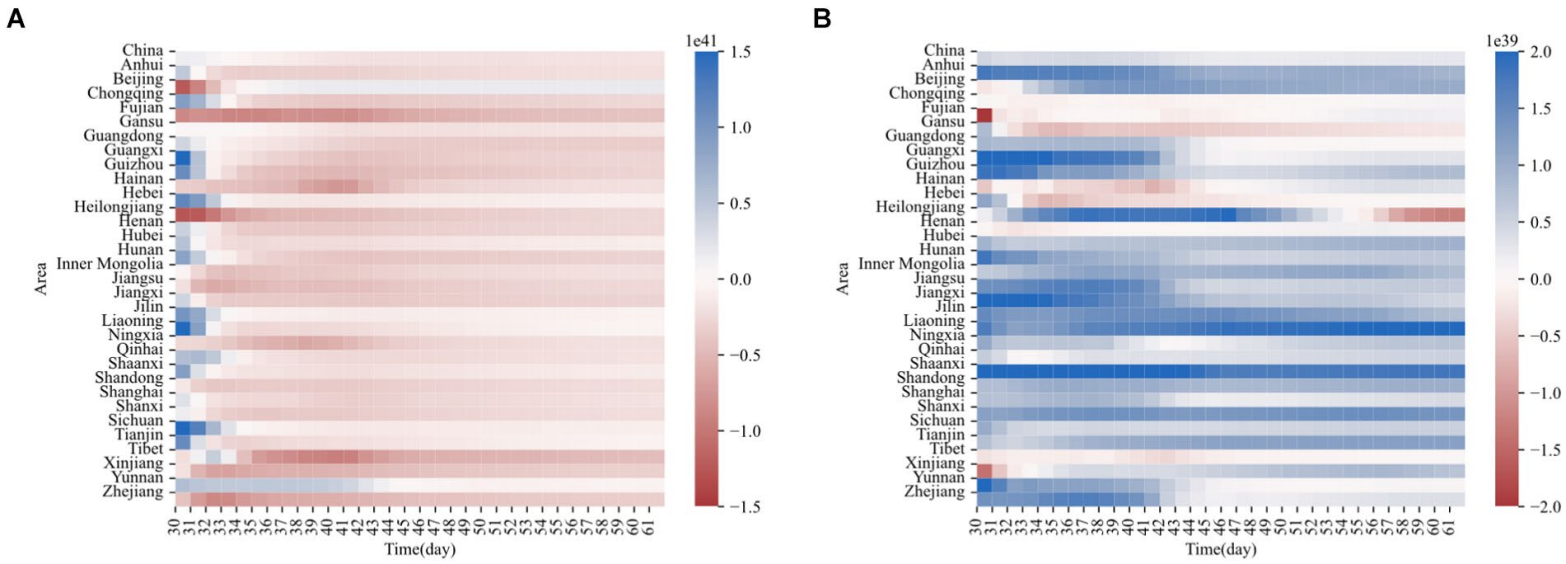
**(A)** Heat map of numerical values for φ₁(T). **(B)** Heat map of numerical values for φ₂(t).

In this context, $\varphi_{1}\left(t\right)=a_{1}x_{2}\left(t\right)+a_{2}{x_{1}}^{2}\left(t\right)$ , and $\varphi_{2}\left(t\right)=b_{1}x_{2}\left(t\right)+b_{2}{x_{2}}^{2}\left(t\right)+b_{3}x_{1}\left(t\right)x_{2}\left(t\right)$ . The magnitude of $\varphi_{1}\left(t\right)$ can gauge the impact of online medical consultation on online medical search at time t , while the magnitude of $\varphi_{2}\left(t\right)$ can measure the influence of online medical search on online medical consultation at time t . We conducted statistical analyses of $\varphi_{1}\left(t\right)$ and $\varphi_{2}\left(t\right)$ for each region between days 30 and 61 and illustrated the results through a heatmap (Figure 6).

Upon examining the heatmap, we were pleasantly surprised to discover common patterns. As time progresses, the impact function $\varphi_{1}\left(t\right)$ of online medical consultation on online medical search tends to show negative values in most regions. Conversely, the impact function $\varphi_{2}\left(t\right)$ of online medical search on online medical consultation generally exhibits positive values in the majority of regions. Notably, when fitting the data solely for Beijing, $\varphi_{1}\left(t\right)$ ultimately remains positive; when fitting the data exclusively for Gansu and Heilongjiang provinces, $\varphi_{2}\left(t\right)$ ultimately remains negative. These results suggest the presence of a predation mechanism between online medical consultation and online medical search, where online medical consultation inhibits online medical search, and online medical search promotes online medical consultation. This phenomenon is akin to a predation mechanism in ecology—online medical consultation’ inhibitory effect on online medical search does not eliminate the latter but rather maintains a relative balance, akin to a coexistence relationship within the information system.

### 5.2 In-depth elaboration of predation mechanisms

**Figure 7:**
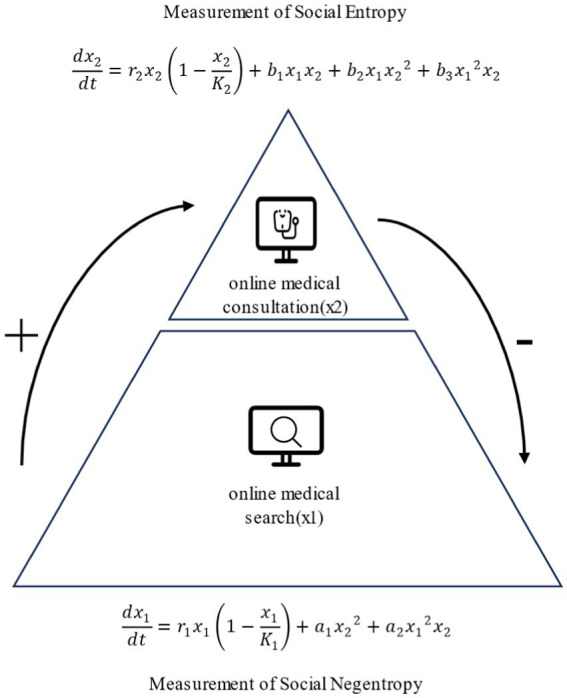
Predation mechanisms in online medical search and online medical consultation.

The predation mechanism between online medical consultation and online medical search is essentially propelled by varying levels of health information needs, as illustrated in Figure 7. Robert S. Taylor posited a discrepancy between perceived needs and expressible needs, categorizing information needs into different levels (45). Health information needs constitute a subset of information needs, and the health information needs of internet users naturally manifest at different hierarchical levels.

In the context of an epidemic, the public perceives a lack of knowledge to cope with the challenges, giving rise to information needs. Due to the convenience of online medical search, individuals often initially seek health information through online searches (46). However, only health information needs that can be articulated in language can be expressed and utilized to retrieve relevant information through search engines. Meanwhile, due to the shortcomings in the accessibility, readability, and comprehensibility of online medical information (10, 47), health information needs expressed clearly may not be fully met by online medical search alone. Many internet users, after conducting online medical search, find their health information needs inadequately addressed. Therefore, after online medical search, they often turn to more trusted sources to obtain health information. Compared to online medical search, patients value healthcare professionals as sources of medical advice (48), and patients typically use online medical search as preparation before consulting a doctor (49). This leads many internet users to turn to online medical consultation after conducting an online medical search, reflected in the big data of internet users’ behavior as an increase in cumulative BESI data promoting an increase in cumulative BHII data.

In addition to the information needs that internet users are aware of and willing to obtain, there are also information needs that users are willing to acquire but may not be consciously recognized. These are referred to as explicit information needs and implicit information needs, respectively (50, 51). The rich experience of healthcare professionals enables them to, through online conversations with users, not only effectively meet users’ explicitly articulated information needs but also assist users in identifying and satisfying implicit information needs that they may not be aware of. In summary, online medical consultation typically effectively address the health information needs of internet users. After consulting healthcare professionals on online platforms, users often do not need to seek health information through online searches. The inhibitory effect of online medical consultation on online medical search is essentially due to a reduction in the number of people with health information needs. This phenomenon is reflected in the big data of internet users’ behavior, where an increase in cumulative BHII data appears to inhibit the increase in cumulative BESI data.

Viewed from the perspective of the social system, the predation of online medical consultation and online medical search driven by health information needs can be seen as a process in which the social system extracts negentropy. The COVID-19 pandemic has brought severe disruption to social order, manifesting not only in the pressures on the healthcare system, social isolation, and material disruptions such as economic recession but also involving emotional and psychological disturbances (52). When studying social systems, the concept of social entropy is commonly employed to describe the degree of disorder and uncertainty in the social environment (53). The chaos in social order caused by the pandemic can be considered an increase in social entropy. Excessive increases in social entropy may lead to the collapse of the social system, requiring negentropy to maintain social stability. Social systems feed on negentropy, extracting it from sources to maintain and construct organized structures (54). Knowledge and information are essential sources of negentropy (55, 56). The social system’s need for negentropy is reflected at the individual level as a need for information.

In different sociological contexts, there are variations in the definition and measurement methods of social entropy (57). The crucial aspect is the ability to effectively describe the degree of disorder and uncertainty in the social environment (53). Numerous scholars in previous research have demonstrated the correlation between the relative frequency of online medical search and the number of patients (41, 42). Therefore, quantified data from online medical search can be used to measure social entropy. Conversely, negentropy represents order (58). Online medical consultation are a crucial means of maintaining social order during a pandemic. On the one hand, online medical consultation alleviates the shortage of medical resources, avoids offline contact (59), and prevents further social disorder. On the other hand, the majority of patients express high satisfaction with online medical consultation (60). Patients often experience recovery through online medical consultation. Hence, quantified data from online medical consultation can be used to measure social negentropy. The predation mechanism between online medical consultation and online medical search is a dynamic process. The promotion of online medical search by online medical consultation can be seen as an increase in social negentropy, while the inhibition of online medical search by online medical consultation can be seen as a decrease in social entropy. The predation mechanism, through the increase in negentropy and the decrease in entropy, drives an overall reduction in social system entropy, reaching an equilibrium state.

### 5.3 Insights

Our research has identified and explained the predation mechanism between online medical search and online medical consultation driven by health information needs. This provides inspiration for better meeting the health information needs of netizens and harnessing the negentropy of medical information.

Firstly, we need to consider how to enhance online medical search to better meet the health information needs of netizens. Nowadays, search engines and online medical consultation are becoming increasingly intelligent. Search engines are proficient in providing personalized search results based on users’ search history, location, interests, and preferences (61). However, excessive personalization may trap netizens in information bubbles (62). Implicit information needs require individuals to explore the information system to understand what information they need. Once these needs are recognized, individuals will choose to satisfy them (63). Therefore, search engines need to recommend information from different perspectives and viewpoints based on users’ searches, rather than recommending information closely related to types and topics. An ideal search engine should serve as a universal knowledge base, allowing users to obtain comprehensive information and meet their multi-level information needs. Secondly, it is crucial to avoid information overload caused by online medical search. Improving the quality of online medical information and filtering out low-quality information should be prioritized. Trustworthy online medical information sources should be prioritized in search results.

Secondly, the predation mechanism between online medical search and online medical consultation implies that we can promote online medical consultation through online medical search. During a pandemic, promoting online medical consultation can effectively alleviate the shortage of offline medical resources and reduce the risk of infection by minimizing offline contact in medical institutions (64). Even in normal circumstances, actively promoting online medical consultation holds significant value. Specialized doctors have limitations in their knowledge, while online medical consultation platforms can facilitate collaborative consultations among experts, providing patients with higher-quality medical advice. Specific implementation strategies include optimizing the quality of online health information to enhance netizens’ trust in online medical search. Secondly, adopting reasonable recommendation strategies to recommend relevant content to users can increase their interest and engagement, leading to higher search volume. Additionally, we encourage doctors and other professionals to actively participate in the development of telemedicine. To promote online medical consultation through the predation mechanism, there must be sufficient capacity for online medical consultation. Using online medical search to promote online medical consultation is a more intelligent and efficient approach than traditional promotional methods. The goal is to leverage the predation mechanism of these two health information-seeking behaviors to establish a highly interconnected medical ecosystem, meeting the growing need for medical services and improving the accessibility and efficiency of medical resources.

Thirdly, we should harness the negentropy of internet medical information. There is an abundance of medical information available on the internet, which may lead to information overload—where the provided information exceeds the searcher’s capacity to obtain and process it (65). Online medical search provide a vast amount of medical information, but the quality of this information varies and may not be suitable for each patient’s specific situation. Moreover, excessive medical information may cause anxiety and panic, especially when individuals incorrectly associate themselves with various diseases (66). Information overload often results in confusion and feeling overwhelmed. Additionally, obtaining specific and real-time medical advice from professional sources through online medical consultation can reduce the uncertainty of the medical information obtained. Therefore, we should enhance the quality of internet medical information, regularly update it, and encourage netizens to obtain medical information from professional sources.

### 5.4 Data scope and study limitations

Our study utilized the “Epidemic Index” launched by Baidu, a leading online platform in China. User behaviors in information searching and sharing provide valuable socio-economic and psychological insights, aiding in understanding regional health trends (67). Consequently, BESI and BHII effectively quantify the evolutionary patterns of online medical search and consultation behaviors in the analyzed regions. Baidu, the predominant search engine in China, had 648 million monthly active users as of December 2022 (68, 69). Data from Baidu’s search engine accurately reflect public medical needs during the pandemic (70), and local search engine data generally offers stronger predictive power than combined indices (71, 72). Moreover, the “Epidemic Index,” an analytical tool developed by Baidu, provides more than just generalized nationwide statistics. This study collected data from 31 provincial-level administrative regions in China, excluding Hong Kong, Macau, and Taiwan, for regional and aggregated national data modeling. Therefore, the study’s data are representative and comprehensive for various regions, populations, and platforms across China.

The data span a period of 2 months, from November 26, 2022, to January 25, 2023, corresponding to a wave of the COVID-19 pandemic. The “Epidemic Index” was introduced following the Chinese government’s release of the “New Ten Measures” aimed at optimizing pandemic-related policies (73). Considering China’s large population and relatively limited medical resources, the government maintained stringent control measures during the COVID-19 pandemic to protect public safety and health (74). However, multiple virus mutations, the reduced severity of the Omicron variant, increased transmissibility, and widespread vaccination have mitigated the overall health risk, necessitating more precise and scientific control measures. As a result, various regions in China optimized their epidemic prevention strategies, adopting more flexible measures, which led to an increase in the number of confirmed COVID-19 cases. Due to the temporary offline status of the “Epidemic Index” and its data coverage of only the preceding 2 months, we collected 2 months of continuous data for our study. Although the data cover only 2 months, this period corresponds to a wave of the COVID-19 pandemic, allowing us to quantify the evolutionary patterns of online medical search and consultation behaviors during this epidemic phase.

Nevertheless, our study may have limitations in explaining health information-seeking behaviors in other regions, populations, and contexts. First, due to the specific nature of our data sources, we were unable to obtain suitable data from other Chinese online platforms, such as the National Health Commission of China or Dingxiang Doctor, or from representative data sources in other countries and regions, such as Google, the World Health Organization, or Johns Hopkins University, that could quantify health information-seeking behaviors representing different levels of health information needs. Consequently, our research focused on exploring the interaction between online medical searches and consultations within the scope of the Baidu platform in China. Investigating the evolutionary patterns of health information-seeking behaviors in other regions, populations, and contexts remains a direction for future research.

Secondly, the generalizability and applicability of our study are primarily demonstrated by the methodology we used to explore interactive relationships. This method is broadly applicable, as evidenced by its effectiveness for data from different time periods. For instance, after the re-launch of the “Epidemic Index,” we collected data from May 6, 2023, to July 4, 2023, when the COVID-19 pandemic had returned to a normalized state and was gradually subsiding. Using the data modeling approach developed in this study, we modeled the “Epidemic Index” for this period, resulting in the following optimal model:


$$\left\{ \begin{array}{c} \frac{dx_{i1}}{dt}=r_{i1}x_{i1}\left(1-\frac{x_{i1}}{K_{i1}}\right)+a_{1}{x_{i1}}^{2}{x_{i2}}^{2}\\ \frac{dx_{i2}}{dt}=r_{i2}x_{i2}\left(1-\frac{x_{i2}}{K_{i2}}\right)+b_{1}{x_{i1}}^{2}{x_{i2}}^{2} \end{array}\right.$$

The parameter meanings are consistent with those in Table 2, with $a_{1}>0$ and $b_{1}>0$ . In our pattern detection experiments, the fit rates for $a_{1}{x_{i1}}^{2}{x_{i2}}^{2}$ and $b_{1}{x_{i1}}^{2}{x_{i2}}^{2}$ were 83.17 and 98.19%, respectively. The differences in experimental results indicate that the evolutionary patterns of online health information-seeking behaviors vary between epidemic waves and periods of normalized epidemic status. This suggests that these phases should be analyzed separately in discussions. The high fit rates also demonstrate that our data modeling method is suitable for studies across different periods. The successful detection of evolutionary patterns in various periods validates our approach, showing that optimal models can be derived for different research subjects using our foundational model. Therefore, the methodology and approach of this study can be extended to further research on health information needs across other platforms, regions, and populations.

Additionally, we applied this method to other datasets, such as COVID-19 case data and Baidu search data, vaccine data and Baidu search data, and data from other fields like entertainment events and movie box office data from Baidu searches and news. These experiments verified the scientific validity and feasibility of our method.

## 6 Conclusion

This study employed data modeling to explore the interplay between two health information-seeking behaviors: online medical searches and online medical consultations. The findings reveal a predatory mechanism driven by varying levels of health information needs between these two behaviors. The main conclusions drawn from this study are as follows:

This study proposes a method to explore the underlying patterns of nonlinear correlations and mutual influences among different behaviors. By selecting appropriate quantified data and constructing precise foundational models to quantify their evolutionary patterns, we define an impact function to measure the direct mutual influences among different behaviors. The mathematical model, capable of quantifying the nonlinear correlations among different behaviors, is then constructed by fitting the data and determining the impact function structure with the maximum commonality. Through in-depth analysis of the model, we can gain insights into the mechanisms of mutual influences among different behaviors.
The study employs the “Epidemic Index” to quantify two health information-seeking behaviors, namely, online medical search and online medical consultation. Using the cumulative BESI and BHII as core variables, the study adopts the modeling approach inspired by the logistic model and validates it. Through fitting 32 sets of data, a mathematical model capable of quantifying the mutual influence mechanism between online medical search and online medical consultation is established.
Through numerical analysis of the model, this study explains the underlying mechanism between online medical consultation and online medical search. Specifically, an increase in online medical search promotes an increase in online medical consultation, but an increase in online medical consultation continuously promotes an increase in online medical search. We delve into this predation mechanism, analyze the driving role of health information needs, and observe the balance between social entropy and negentropy reflected by the predation mechanism.
Based on our research, we present insightful recommendations to better meet the public’s health information needs and leverage the negative entropy of internet medical information. These suggestions include improving online medical search to better fulfill the health information needs of netizens, utilizing the predation mechanism between online medical consultation and online medical search to promote online medical consultation, and enhancing the quality of internet medical information while encouraging netizens to obtain health information from professional sources. These recommendations contribute to better-meeting people’s health information needs and reducing the increase in social entropy, particularly during unforeseen events such as pandemics.
While the study successfully modeled the predatory mechanism between online medical searches and consultations using data from the Baidu platform, there are unresolved issues regarding the generalizability of these findings to other regions, populations, and health information-seeking behaviors. Future research will explore the applicability of the proposed methodology to different platforms, regions, or populations with varying health information-seeking behaviors to broaden the understanding of these interactions.

## Data Availability

The original contributions presented in the study are included in the article/Supplementary material, further inquiries can be directed to the corresponding author.
